# Oroesophageal Pemphigus vulgaris Secondary to Lisinopril Use: A New Side Effect

**DOI:** 10.7759/cureus.14333

**Published:** 2021-04-06

**Authors:** Usman Tariq, Adeel Nasrullah, Aritra Guha, Marcia Mitre

**Affiliations:** 1 Internal Medicine, Allegheny Health Network, Pittsburgh, USA; 2 Gastroenterology, Allegheny Health Network, Pittsburgh, USA

**Keywords:** pemphigus vulgaris, pemphigus foliaceous, drug allergy

## Abstract

Pemphigoid diseases are a group of blistering autoimmune pathologies including pemphigus vulgaris (PV) and pemphigus foliaceous, which affect mucocutaneous tissues. Non-steroidal anti-inflammatory drugs, penicillamine, and angiotensin-converting enzyme inhibitors such as captopril and enalapril are associated with drug-induced pemphigoid. We present a case of lisinopril-associated PV which has not been previously reported.

## Introduction

Pemphigus diseases (PD) are a group of blistering pathologies caused by an autoimmune response to structural proteins called desmogleins (DG) that allow for cell-cell adhesion within the skin and mucous membranes. A targeted attack on adhesive proteins results in the loss of connection between keratinocytes, a process known as acantholysis, leading to the development of intraepithelial blisters in affected tissues. Based on varying clinical, histological, and immunological presentations, PD are subdivided into four subtypes, including pemphigus vulgaris (PV), pemphigus foliaceous (PF), paraneoplastic pemphigus, and immunoglobulin (Ig) A pemphigus [1]. Precipitating factors of PD include genetic predisposition, environmental agents, and drugs [2-4]. Drug-induced PD (DIP) is a well-known entity that is attributed to multiple agents such as non-steroidal anti-inflammatory drugs (NSAIDs), penicillamine, and angiotensin-converting enzyme inhibitors (ACEI) [5]. Among ACEI, commonly implicated agents include captopril and enalapril [6-9]. Previous cases of lisinopril-induced PD have been reported in the literature [10]. However, per our literature review, a lisinopril-induced PV has not been previously reported.

This article was previously presented as a meeting abstract at the American College of Gastroenterology 2020 Annual Meeting on October 26, 2020 [11].

## Case presentation

**Figure 1:**
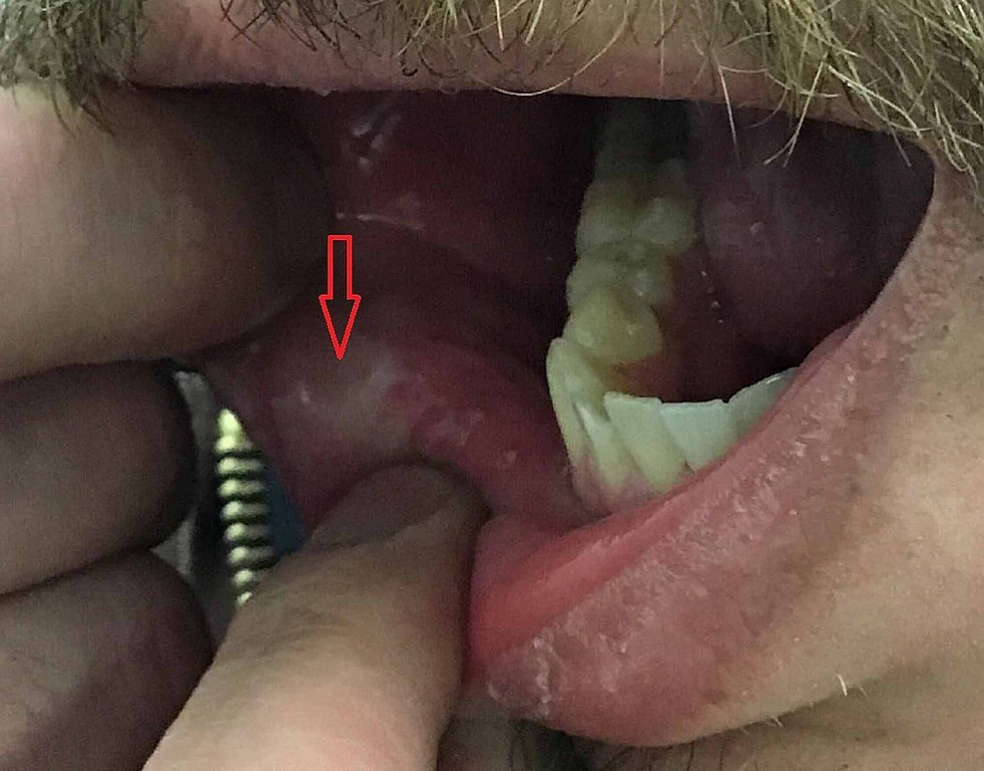
Red arrow pointing towards a white-colored, shallow oral ulcer along the buccal mucosa.

A 35-year-old male with a past medical history of essential hypertension presented with oral pain, dysphagia, odynophagia, and a concomitant 20-lb weight loss in six weeks following initiation of lisinopril 20 mg once daily (OD) by his primary care physician. The patient denied a concomitant history of fatigue, low-grade fevers, photosensitivity, nasal/nasopharyngeal ulcerations, malar/discoid rash, arthiritis, artharalgias, mylagias, pleuritic/inspiratory chest pain, easy bruising, recurrent infections, anemia, seizures, or psychosis. His past medical history was also negative for PD, allergies, and autoimmune disease(s). He also denied recent use of NSAIDs, penicillamine, previous use of captopril, enalapril, or over-the-counter herbal supplements. On physical examination, multiple white-colored, shallow oral ulcers were noted along the buccal mucosa (Figure 1). A pale white-colored discharge was also noted along the buccal mucosa. A psychological examination was negative for acute mental status changes. A normal S1/S2 without murmurs, rubs, or gallops were noted. A thorough dermatological examination was negative for any recent skin and soft tissue changes.

**Figure 2:**
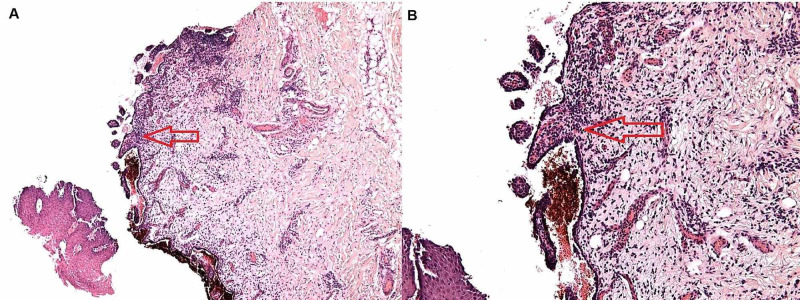
Red arrows depicting mucosal epithelium with a suprabasal split associated with intraepithelial acantholysis and an interstitial lymphoplasmacytic infiltrate, (A) ×20 (B) ×40 magnification.

A complete blood count (CBC) with differentials and comprehensive metabolic panel (CMP) was within normal limits. Tests for anti-nuclear antigen [11] antibody, human immunodeficiency virus, hepatitis B virus, and hepatitis C virus were negative. Due to concerns for oral candidiasis, he was prescribed fluconazole (200 mg on day one, followed by 100 mg OD for seven days) without resultant improvement. As such, he was transitioned to valacyclovir 1,000 mg TID a day for seven days; however, he continued to complain of persistent symptoms. He subsequently underwent an intralesional incisional biopsy of the right buccal mucosa which exhibited a mucosal epithelium with suprabasal split, associated intraepithelial acantholysis, and an interstitial lymphoplasmacytic infiltrate (Figure 2).

**Figure 3:**
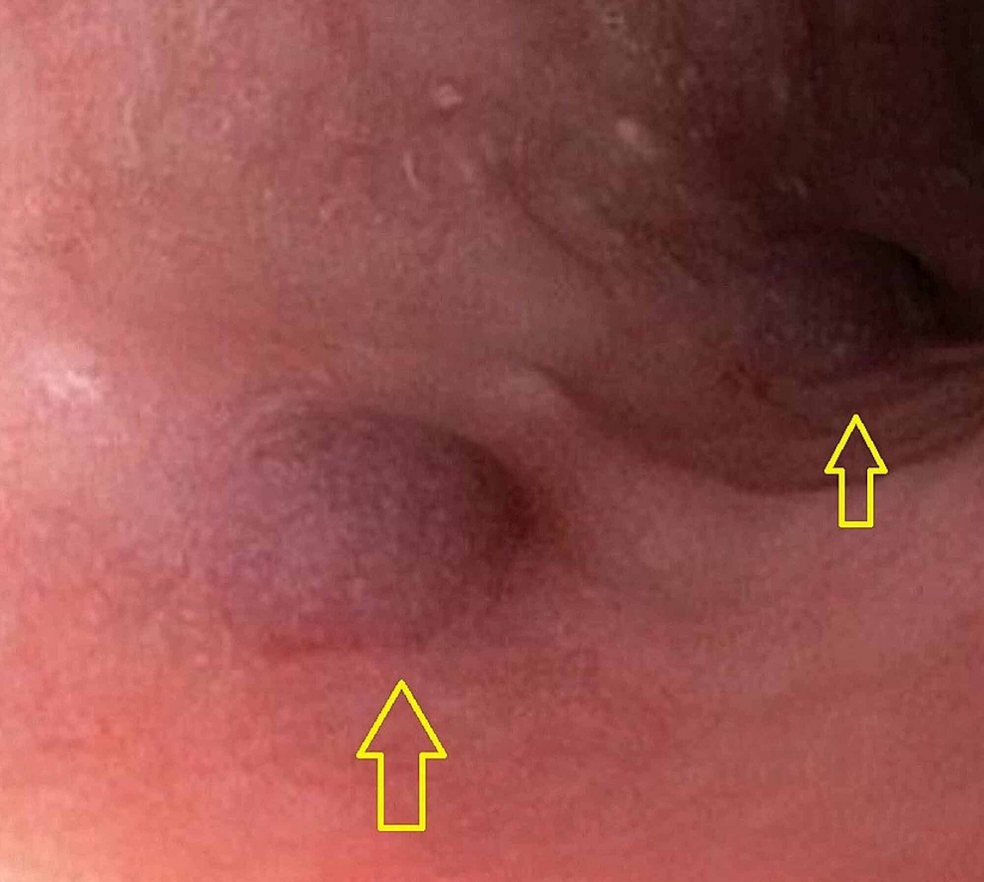
Yellow arrows pointing to two blood-filled blisters in the mid-esophagus seen during EGD. EGD, esophagogastroduodenoscopy

Direct immunofluorescence of a perilesional biopsy specimen demonstrated positive immunoreactivity for IgG in an intraepithelial (chicken-wire-like) staining pattern. There was negative immunoreactivity for IgA, IgM, and complement C3. As such, a diagnosis of drug-induced PV (DIPV) was established. For his dysphagia and odynophagia, resultant esophagogastroduodenoscopy (EGD) showed two blood-filled blisters in the mid-esophagus (Figure 3).

As a diagnosis of PV was previously established, these lesions were not biopsied. The patient’s lisinopril was discontinued and he was started on a prednisone taper, starting at 50 mg OD for two weeks with recommendations to decrease by 10 mg every two weeks. Following this regimen, the patient noted mild improvement in his dysphagia and odynophagia; however, his oral lesions continued to persist. He was recommended to undergo a repeat EGD for subsequent follow-up of his esophageal lesions but denied this intervention. He was then started on mycophenolate 500 mg OD which was increased periodically to a current dosage of 1,500 mg TID for maintenance.

He is currently being followed up as an outpatient with monthly CBC and CMP with plans to repeat DG antibodies every six months till a complete resolution of his symptoms.

## Discussion

PV is characterized by flaccid, mucous, or mucocutaneous blisters with antibodies against DG1 and DG3. The oral mucosa is involved in invariably all cases, while cutaneous lesions may target the scalp, trunk, and intertriginous areas. PF is a less severe variant of PD and only affects the cutaneous tissues with autoantibodies directed against DG1. The specificity of IgG autoantibodies varies considerably in patients with PV [1]. In our patient, a lack of IgA, IgM, and complement activation on direct immunofluorescence indicated an absence of an immune-mediated response. As such, given that our patient was newly exposed to an ACEI and their concomitant association with DIP is well recognized, a diagnosis of DIPV secondary to lisinopril use was established.

The pathogenesis of DIPV varies according to the type of perpetuating agent. Captopril and enalapril are attributed as leading causes of PV and PF among ACEI [7,8]. Captopril contains an active thiol group which is considered acantholytic and can directly lead to loss of cell-cell adhesion [12]. This is thought to be achieved by thiol-mediated activation of plasminogen activator as well as the formation of thiol-cysteine bonds instead of cysteine-cysteine bonds that promote cellular adhesion. Non-thiol drugs such as lisinopril can also trigger DIP; however, this can be attributed to the amide group within their structure. While studies have noted the presence of amide groups in offending agents, a causality to DIP has not been previously established [13,14]. In-vitro studies have noted that lisinopril’s amide group can lead to the alteration in the proteins of the lamina lucida, a component of the oral mucosa’s basal lamina, leading to the formation of autoantibodies against these “neo-antigens” that can be associated with acantholysis [15].

A thorough evaluation of the patient’s medication history is pivotal for diagnosis with the cessation of the offending agent as the mainstay of therapy [16]. Systemic steroids with or without rituximab are first-line treatment strategies [17]. Azathioprine or mycophenolate can be used in patients where long-term glucocorticoid use is contraindicated [18].

## Conclusions

Lisinopril-induced PV is an exceedingly rare side effect of the commonly used antihypertensive. A patient presenting with oral lesions of unclear etiology with infectious and non-infectious causes ruled out and not responding to standard treatment should be evaluated thoroughly for medication history for establishing the diagnosis along with a biopsy of the lesion. The cessation of the drug is the mainstay of treatment along with systemic steroids and immunomodulators.
